# The Influences of Various Housing Systems on Growth, Carcass Traits, Meat Quality, Immunity and Oxidative Stress of Meat-Type Ducks

**DOI:** 10.3390/ani10030410

**Published:** 2020-03-02

**Authors:** Mahmoud M. Abo Ghanima, Mohamed A. El-Edel, Elwy A. Ashour, Mohamed E. Abd El-Hack, Sarah I. Othman, Maha A. Alwaili, Ahmed A. Allam, Asmaa F. Khafaga, Ayman H. Abd El-Aziz

**Affiliations:** 1 Animal Husbandry and Animal Wealth Development Department, Faculty of Veterinary Medicine, Damanhour University, Damanhour 22511, Egypt; eledel.m@vetmed.dmu.edu.eg (M.A.E.-E.); ayman.sadaka@vetmed.dmu.edu.eg (A.H.A.E.-A.); 2 Poultry Department, Faculty of Agriculture, Zagazig University, Zagazig 44511, Egypt; dr.elwy.ashour@gmail.com; 3 Biology Department, College of Science, Princess Nourah bint Abdulrahman University, Riyadh 11671, BO. Box 24428, Saudi Arabia; sialothman@pnu.edu.sa (S.I.O.); maalqaraawi@pnu.edu.sa (M.A.A.); 4 Department of Zoology, Faculty of Science, Beni-suef University, Beni-suef 65211, Egypt; allam1081981@yahoo.com; 5 Department of Pathology, Faculty of Veterinary Medicine, Alexandria University, Edfina 22758, Egypt; Asmaa.Khafaga@alexu.edu.eg

**Keywords:** Pekin ducks, housing system, meat cholesterol, meat triacylglycerol, oxidative stress

## Abstract

**Simple Summary:**

The present investigation aimed to examine the impacts of different housing systems on growth performance traits, carcass characteristics, meat quality criteria, immunity, and oxidative stress of meat-type ducks. The study concluded that housing Pekin ducks in a house with a yard and a swimming pool positively affected growth performance traits, carcass characteristics, meat quality, blood lipid profile, immunity, as well as blood antioxidative status.

**Abstract:**

The present study aimed to investigate the effect of different housing systems on Pekin ducks. A total of 300-day old Pekin ducklings were randomly divided into four experimental groups; the first housed in a closed house (CH), the second in closed house with open yard (HY), the third group in closed house with swimming pool (CHSP) and the fourth in a closed house with swimming pool and yard (HYSP). Results indicated that the HYSP and CHSP produced higher body weight comparing to the other groups. However, the HYSP gave the highest body weight followed by CHSP then HY and CH. The same trend was observed regarding weight gain and feed-conversion ratio (FCR). Moreover, HYSP, HY and CHSP showed higher dressing percentage, breast muscles and thighs and lower abdominal fat than the CH group. Serum protein was significantly higher in HYSP and HY than that of the closed house. While, lipids, cholesterol and triacylglycerol were significantly lower in groups housed in HY than that of CH. Meat cholesterol and triacylglycerol reduced in groups reared in HY. Housing ducklings in yards and using swimming pools significantly improved the general immunity (phagocytic index and activity and differential leucocytes count), and also improved the oxidative stress parameters. In conclusion, results confirmed that housing ducks in a house supplied with yard and swimming pool can improve its productivity, carcass traits, meat quality, blood lipid profile, immunity and antioxidative status.

## 1. Introduction

Waterfowls could be applied to resolve the lack of animal protein in the human diet. They differ from the other livestock types in some characters; they grow fast, particularly during the early stage of life, with a relative high efficiency in feed conversion. Moreover, they do not need a high proportion of protein in their feed, in contrast to the other poultry species. Ducks live happily under a wide range of climatic conditions and they are free from common poultry diseases, such as leucosis, Marek’s disease, infectious bronchitis and other respiratory troubles [1,2,3,4,5]. The duck, as a waterfowl, has a different physiology than the other poultry types, where their breast muscles have a higher red-fiber content than chickens [6], and so are considered as a red meat source. Moreover, despite the high-nutritive value of duck meat, the easiness of raising and the less susceptibility to many of the common poultry diseases, the high-fat content in duck meat is the hidden reason for the unacceptability of the duck meat by a proportion of consumers [7].

Compared to turkey or hen meat, duck flesh has higher lipid content and oxidative energy metabolism [8]. Because of the high-fat content in duck meat, an alternation occurs in these fats over the storage time and affects the physicochemical and sensory properties in the form of raw meat or processed products [9]. Additionally, the high-fat content in duck meat leads the meat to be easily oxidized by oxygen and to contain a stronger odor compared to chicken meat. Odor sensation, which originates from thousands of low-molecular weight compounds, consists of aliphatic and aromatic compounds that normally contain a heteroatom [10].

The majority of commercial duck farming in Egypt depends on closed-farm rearing, either naturally ventilated or controlled houses, or these types of farms may not be able to provide a high-level of welfare for ducks as waterfowls. Many researches have stated the desirable effects of providing access to water for ducks, as it improved the health condition and was seen to benefit eye and feather cleanliness and foot pad condition [11,12,13,14]. Other investigators have reported the increase in meat production in ducks allowed to access a free-range environment and water [15].

Many studies suggested that rearing method is one of the numerous non-genetic agents that can greatly affect meat goodness and carcass traits [1,16]. Intensive duck farming has reduced the total cost of production of duck meat; however, that intensification has adverse effects on the quality of the product quality and leads to the deteriorated welfare of the birds, as ducks kept in intensive farming systems possess many disorders, including health problems and behavioral alternation, which cause self-mutilations and cannibalism [17]. Thus, for producing duck meat of higher quality, ducks need to be kept under environmental and management conditions that ensure the provision of a reasonable level of welfare [18], as duck meat quality, duck fattening performance and duck welfare are largely affected by the environment and rearing system [17].

Controlling the duck house environment in closed houses is very difficult and requires adequate ventilation, with consideration to farm temperature and other management indices [14]; therefore, there is a need for the provision of more information about the productivity of ducks in differing hosing environments; comparing a closed system with a yard, and with the presence or absence of access to water for swimming. The current study investigates the effect of different housing systems for ducks in order to provide sufficient evidence on the best system for ideal growth performance, carcass traits, meat quality, immunity and antioxidative stress.

## 2. Materials and Methods

All investigations in this study were carried out in accordance to the Native Experimental Animal Care Committee and approved by the Ethics of the Institutional Committee of Animal Husbandry and Animal Wealth Development Department, Faculty of Veterinary Medicine, Damanhour University, Egypt (DMU/VetMed-2019-/0145).

Three hundred one-day-old Pekin ducklings were used in the current study and were randomly divided into four groups with five replicates in each one (4 × 5 × 15). Ducklings were obtained from a local company (El Fashny in Damanhur city, El Behira, Egypt). Birds were floor brooded for the first week, using supplemental feeders and drinkers, in a closed house, in separate partitions for each replicate. From the second week, ducklings of the housing system which included a yard were allowed to go out into an attached yard for seven hours daily during the daylight time (9 am to 4 pm), then for 10 h daily from four weeks of age (9 am to 7 pm). Two groups—one housed in the closed house and one housed in the closed house with attached yard—were supplied with a swimming pool for each replicate, and the swimming pool was filled with water from the second week of age. The space for the group housed in the closed house (CH) was 15 m^2^, which was divided into five small separate pens for each replicate of 3 m^2^. The group housed in the closed house with a yard (HY) were housed in total space of 30 m^2^, divided into five pens, one for each replicate, and each pen was 6 m^2^—a 3 m^2^ closed pen attached to a small yard of 3 m^2^. The group in the closed house with swimming pool (CHSP) was divided into five separate pens, with a total floor area of 25 m^2^, each one included a small swimming pool of 2 m^2^ and a 3 m^2^ floored area (5 m^2^ per pen). The group housed in the closed house with yard and swimming pool (HYSP) was housed in a total area of 40 m^2^, divided into five separate areas, each area of 8 m^2^, divided into a 3 m^2^ floored area of the house with a 2 m^2^ swimming pool and attached to a 3 m^2^ yard (see the supplementary materials). Chicks were floor brooded at 33 °C at the birds’ level during the first three days of age, and then the temperature was gradually reduced to reach the room temperature (24 °C at 14 days of age. Birds were subjected to the recommended vaccination programs. Ducks were fed *ad-libitum* on Feed mix duck starter diet (Feed mix 711) (22% protein and 2900 kcal/kg) from one to seven days of age, then fed on Feed mix duck grower diet (Feed mix 712) (18% protein and 2900 kcal/kg); manufactured by Feed mix Egypt for the feed industry, the extension of first industrial area No. 12008, El Obour city, Cairo, Egypt. The experiment was performed from the middle of March to the middle of May; this time of year, according to the local environmental conditions of Egypt, is characterized as being a good rearing environment, with ambient temperatures ranging from 18 to 25 °C and with minimal air drafts.

### 2.1. Estimation of Growth Traits

The investigated growth traits included body weight (BW), weight gain (WG) and feed-conversion ratio (FCR). For BW, ducks were individually weighed from the beginning of the experiment (one-day old) by a weekly interval until eight weeks of age. The weighing of birds was done every week in the early morning, before receiving any feed or water. Weekly WG of birds was calculated by subtracting the body weight of the bird at a certain week, from the body weight of the same bird at the next week. The FCR was calculated by dividing the amount of feed intake (g) during the week by the gain in weight (g) during the same week [19].

(1) $$\mathrm{FCR}=\;\frac{\mathrm{feed}\;\mathrm{intake}\;\left(g\right)/\mathrm{bird}/\mathrm{week}}{\mathrm{weight}\;\mathrm{gain}\;\left(g\right)/\mathrm{bird}/\mathrm{week}}$$

### 2.2. Carcass Traits

Prior to slaughtering, birds (10 birds from each group) were deprived of feed for 12 h then weighed. After slaughtering, birds were scalded, wet-plucked and eviscerated. Then, technological division of the carcass was performed and calculated according to Wang [20]. The carcass was separated to the following cuts: Breast, including the sternum and breast muscles; thigh, weighing two thighs and taking average; shoulder, weighing two shoulders and taking the average; left filet, the de-skinned left breast muscle on the left side of sternum. Liver, heart and gizzard were separately weighed to determine the dressed weight and the dressed percentage. The blood, viscera, lungs, limbs, head and neck were termed as offal and discarded. The abdominal fats in the pelvic and abdominal cavity were completely collected from the carcass and weighed.

### 2.3. Blood Biochemical Parameters

A total of 25 blood samples were collected from each group (five samples from each replicate) from the wing vein on day 56 of the experiment. After collecting blood samples, tubes were left in a slope position till serum samples were separated through centrifugation at 3000 rpm for 15 min. The sera were collected and preserved in a deep freezer at −20 °C until the time of analysis. Serum total lipids were determined by the total lipid kit of Bio-diagnostic according to the method of Zollner and Kirsch [21]. Triacylglycerol was determined using the triacylglycerol kit of Bio-diagnostic according to the method of Fossati and Prencipe [22]. Cholesterol level was determined as described by Allian et al. [23]. The concentration of total protein, albumin and globulin were determined according to the methods of Gornal et al. [24], Doumas et al. [25] and Coles [26], respectively.

### 2.4. Meat Content of Triacylglycerol and Cholesterol

Samples collection, storage and preparation of extract were done according to Folch et al. [16]. Meat triacylglycerol was determined by the triglyceride kit of Bio-diagnostic according to Fossati and Principe [27]. Whereas meat cholesterol was determined using the cholesterol kit of Bio-diagnostic as described by Richmond [28] and Allain et al. [23].

The oxidative stress parameters, including malondialdehyde (MDA), glutathione peroxidase (GPx) and superoxide dismutase (SOD), were measured using spectrophotometry by the methods of Jo and Ahn [29], Paglia and Valentine [30] and Martin Jr., et al. [31], respectively.

### 2.5. Phagocytic Index, Phagocytic Activity and Cellular Immunity

The phagocytic action and phagocytic indicator were determined according to Kawahara et al. [32]. Fifty µg of *Candida albicans* culture was added to 1 mL of citrated blood from each sample, and incubated in water bath at 25 °C for five hours, and then blood smears from each tube were stained with Giemsa stain. Phagocytosis was estimated by determining the proportion of macrophages that contained intracellular yeast cells, in a random count of 300 macrophages, and expressed as percentage of phagocytic activity (PA). The number of phagocytized organisms was counted in the phagocytic cells and called the phagocytic index (PI).

Phagocytic activity (PA) = Percentage of phagocytic cells containing yeast cells.

(2) $$\mathrm{Phagocytic}\;\mathrm{index}\;\left(\mathrm{PI}\right)\;=\;\frac{\mathrm{Number}\;\mathrm{of}\;\mathrm{yeast}\;\mathrm{cells}\;\mathrm{phagocytized}}{\mathrm{Number}\;\mathrm{of}\;\mathrm{phagocytic}\;\mathrm{cells}}$$

For the differential leucocytic count, blood film was produced according to the mode reported by Lucky [33] for each sample. Ten drops from May-Grunwald stain stock sol on a curt, unfixed smear were added to an equal amount of dstilled water, then mixed and left for one minute for staining. The dye was decanted ready for rinsing. Diluted Giemsa’s solution (10 drops of the dye were added to 10 mL of distilled water) was poured over the film as counter stain and left for 20 min, then rinsed in water current and examined by oil immersion lens. The percentage and absolute value for each type of cell were calculated according to Schalm et al. [34].

### 2.6. Statistical Analysis

Data were analyzed by statistical analysis system [35], one-way analysis of variance, Proc. GLM with the following model: X_ijkl_ = μ + A_j_ + e_i_,

where:

X_ij_ An observational data;
μ Overall mean;
A_j_ Effect of housing system;
e_i_ Random error.

## 3. Results and Discussion

Data presented in Table 1 showed that the group housed in a HYSP had a significantly higher average body weight (*p* ≤ 0.001) from the third week of age and in most of the experiment weeks, followed by the group housed in a HY, then the group a CHSP, but the difference was not significant. However, the group housed in a CH showed significantly lower body weights than the other groups. The final average weight at 56 days of age showed slightly different results, as all the groups showed a significant difference, with the following order, from the highest, HYSP then CHSP then HY groups, and the lowest final average body weight was seen in the ducks in the CH group (3901.47, 3788.87 and 3735.93 vs. 3682.47, respectively. The obtained data suggested that housing ducks in a house with yard and presence of a swimming pool may have a desirable effect on body weights. These findings indicate the nature of ducks as waterfowl, thus allowing access to water and an open yard improves ducks’ welfare and fattening performance.

Similarly, data obtained for weekly WG in Table 1 revealed that the HYSP group had significantly higher WG, followed by the HY group then the CHSP group. The lowest weights were noticed in the CH group during the second week (384.67, 375.20, 365.00 and 364.53, respectively). During the third week, the results were slightly different, as the HYSP group’s weight gain was significantly higher than the CH group (457.20 vs. 429.80), and also higher than HYSP and HY groups, but the difference was not significant. For the fourth week, the HYSP group had higher WG than the HY group, but it was not significant; however, they were significantly higher than that of the CHSP and CH groups (672.53 and 664.67 vs. 639.13 and 618.93, respectively). During the fifth week, the HYSP group showed statistically higher WG; however, the HY, CHSP and CH groups were not significantly different. During the eighth week, the HYSP and CHSP groups were not significantly different in WG; however, they were significantly higher than the HY and CH groups (441.07 and 433.33 vs. 368.20 and 384.73, respectively). The total WG during the experimental period was significantly higher in the HYSP group than the CHSP, HY and CH groups (3857.67, 3745.60, 3692.27 and 3639.07, respectively). The feed consumption was slightly (insignificantly) higher in the CHSP and HY groups than the CH and HYSP groups (Table 1). Data presented in Table 1 showed that FCR had significantly reduced in the HYSP group than the CHSP, HY and CH groups during all experimental weeks, from the second week. The total FCR also revealed similar results, as the HYSP group gave significantly lower FCR than the CHSP, CH and HY groups (2.62, 2.77, 2.77 and 2.78, respectively.) These findings may be attributed to the nature of ducks as waterfowl and the presence of a swimming pool which enhanced the growth performance. This may also explain the increased productivity in the HYSP and CHSP groups over the HY and CH groups. Additionally, the environmental enrichment provided by allowing ducks to have a yard of a suitable area, without allowing ducks to lose energy in a free-range environment, may be the main reason for the desirable improvement in growth traits in the HYSP group. These findings are in agreement with those obtained by Erisir et al. [1], who concluded that using a swimming pool significantly increased the final weights and improved the feed efficiency and weight of Pekin ducks. However, authors reported slightly lower body weights compared to the present studies at six weeks of age. Meanwhile, Damaziak et al. [36] reported different results. Results of the aforementioned authors are in agreement with the present study for the first six weeks, as the ducks reared in outdoor system showed higher weight gain and better FCR than the intensive rearing system in the closed house. But they reported different findings from the seventh week, as the intensive rearing had higher weight gain and better FCR than yard-reared ducks. In addition, the ability of Pekin ducks for to quickly adapt to yard and free-range systems produced higher productivity [37]. Similar results were also obtained by El-Edel et al. [38], who reported an improvement in the outdoor rearing of ducks with respect to growth, WG and FCR.

As shown in Table 2, the HYSP group had significantly higher dressing percentage than that of the CH group (62.40% vs. 60.00%, respectively). Liver percentage was significantly higher in the HYSP, HY and CHSP groups than that of the CH group (4.50%, 4.28% and 4.42% vs. 4.14%, respectively). The abdominal fat percentage was significantly reduced in the HYSP, HY and CHSP groups than that of the CH group (2.02%, 2.17% and 2.11% vs. 2.36%, respectively). Values of breast and thighs percentages were statistically higher in the HYSP group than the CHSP and HY groups; however, they were significantly different than the CH group. The left filet value was significantly higher in the HYSP group than the CH group (12.22% vs. 11.76%, respectively). Gizzard, heart and shoulder data were not significantly different among all groups. The significant improvement in dressing percentage and muscle yield may be due to the great impact of yard rearing on muscle development. Using a free-range environment contributed in increasing muscle fiber diameters. The reduction that occurred in the abdominal fat percentage is due to the increased physical activity in yard-reared ducks. Similarly, Erisir et al. [1] confirmed the desirable effect in improving carcass traits in the experimental groups using swimming pools. Furthermore, Damaziak et al. [36] demonstrated that rearing birds in outdoor yards improved dressing percentage in duck males, but females showed no significant improvement. However, Kolluri et al. [39] reported that rearing ducks in a free-range system slightly reduced the dressing percentage and some carcass traits. The latter authors reported similar findings to ours that yard rearing significantly reduced the abdominal fat percentage. The improvement in muscle fiber diameter in ducks was reported by Gille and Salomon [40], who reported that ducks are characterized by a more intensive growth of limbs after hatching, and that keeping ducks outdoors predisposes them to increased physical activity.

The data of blood biochemical parameters are presented in Table 3. Results showed that the HYSP and HY groups had significantly higher serum albumin than the CHSP and CH groups (2.62 and 2.60 vs. 1.91 and 1.88 mg/dL, respectively). Data of serum globulin and total protein showed the same trend. While, A/G ratio was significantly higher in the HYSP group, with no significant difference among the other groups. The blood glucose was significantly higher in the HYSP and HY groups than the CHSP and CH groups. Data of the lipid profile revealed different findings. Serum total lipids, triacylglycerol and cholesterol were significantly higher in the CH and CHSP groups than the HYSP and HY groups.

In the same way, data presented in Table 3 showed that housing Pekin ducks in a HYSP significantly reduced muscle triacylglycerol and meat cholesterol, than a CHSP, a HY and a CH. These findings are similar to those obtained by Erisir et al. [1], who reported a reduction in serum triacylglycerol and cholesterol in groups reared in an extensive system with a yard than in an intensive system in a closed house. This reduction may be attributed to the increased physical activity in ducks allowed to access a yard and a swimming pool. Similarly, Dong et al. [41] reported that a reduction in serum triacylglycerol and cholesterol of Xianju chickens was due to yard rearing.

The phagocytic index was increased in the HYSP group compared to the CHSP, HY and CH groups and, also, phagocytic activity increased in the HYSP group compared to the CHSP, HY and CH groups (Table 4). Data of the differential leucocytes count revealed significant increase in eosinophil’s percentage, heterophiles percentage, basophiles percentage and monocytes percentage, while lymphocytes percentage showed no significant differences among the groups. The improvement in immunity by housing ducks in a yard, especially with free access to a swimming pool, may be attributed to the environmental enrichment of ducks and the suitability of the addition of access to a swimming pool in relation to their normal behavior as a waterfowl. Contrarily, El-Edel et al. [38] reported lower phagocytic index and activity in outdoor-reared ducks compared to indoor-reared ducks, but this may be due to complete outdoor rearing.

Oxidative stress is usually resulting from the increased production of free-radicals and/or a decrease in the antioxidant mechanism [42,43,44]. Data in Table 5 showed that MDA was significantly reduced in HYSP than in CHSP and HY (1.58 vs. 2.10 and 1.90 nmol/mL, respectively) and it was highest in CH housed ducks (2.54 nmol/mL). The GPx results revealed that HYSP and HY ducks were significantly lower than CHSP and CH. Also, the SOD was significantly reduced in HYSP than CHSP and HY (52.60 vs. 69.20 and 73.20 U/gHb respectively). The highest SOD value was observed in CH housed ducks (90.60 U/gHb).

## 4. Conclusions

It could be concluded that housing ducks in a house with a yard can improve their growth performance, feed-conversion rate and weight gain. Additionally, providing ducks with a swimming pool, either in a closed or a yard system, can enhance their growth performance criteria. Including both yard and a swimming pool in the duck-rearing system is a good way to improve carcass traits and reduce meat and serum lipids, cholesterol and triacylglycerol, as well as to improve the general immunity and oxidative stress parameters in meat-type ducks.

## Figures and Tables

**Table 1:** Effect of different housing systems on the productive performance of Pekin ducks.

Items	Housing System	Day Old	7 Days	14 Days	21 Days	28 Days	35 Days	42 Days	49 Days	56 Days
BW (gm)	CH	43.40 ± 0.32	217.67 ± 1.53	582.67 ± 5.22 ^b^	1012.47 ± 5.86 ^c^	1631.40 ± 5.50 ^d^	2280.93 ± 6.71 ^d^	2881.27 ± 10.10 ^c^	3297.73 ± 6.38 ^c^	3682.47 ± 11.37 ^d^
	HY	43.67 ± 0.32	219.67 ± 2.15	594.87 ± 3.36 ^a^	1039.73 ± 8.68 ^b^	1704.40 ± 5.50 ^b^	2355.33 ± 11.56 ^b^	2937.40 ± 10.14 ^b^	3367.73 ± 11.63 ^b^	3735.93 ± 19.03 ^c^
	CHSP	43.27 ± 0.41	216.33 ± 2.04	580.87 ± 3.46 ^b^	1030.27 ± 6.71 ^bc^	1669.40 ± 5.50 ^c^	2318.13 ± 6.46 ^c^	2933.07 ± 13.57 ^b^	3355.53 ± 11.85 ^b^	3788.87 ± 8.88 ^b^
	HYSP	43.80 ± 0.34	220.00 ± 1.83	604.67 ± 4.48 ^a^	1061.87 ± 7.01 ^a^	1734.40 ± 5.50 ^a^	2435.07 ± 11.36 ^a^	3034.20 ± 21.57 ^a^	3460.40 ± 13.41 ^a^	3901.47 ± 11.06 ^a^
WG (gm)	Housing System	1–7 day	7–14 day	14–21 day	21–28 day	28–35 day	35–42 day	42–49 day	49–56 day	Total gain
	CH	174.27 ± 1.23	365.00 ± 4.83 ^b^	429.80 ± 8.51 ^b^	618.93 ± 7.37 ^b^	649.53 ± 7.37 ^b^	600.33 ± 8.44	416.47 ± 9.36	384.73 ± 8.07 ^b^	3639.07 ± 11.42 ^d^
	HY	176.00 ± 1.91	375.20 ± 3.67 ^ab^	444.87 ± 8.72 ^ab^	664.67 ± 8.18 ^a^	650.93 ± 12.95 ^b^	582.07 ± 12.41	430.33 ± 12.99	368.20 ± 14.17 ^b^	3692.27 ± 18.95 ^c^
	CHSP	173.07 ± 1.63	364.53 ± 4.35 ^b^	449.40 ± 7.87 ^ab^	639.13 ± 7.72 ^b^	648.73 ± 6.91 ^b^	614.93 ± 16.79	422.47 ± 14.80	433.33 ± 16.49 ^a^	3745.60 ± 8.88 ^b^
	HYSP	176.20 ± 1.55	384.67 ± 5.41 ^a^	457.20 ± 7.58 ^a^	672.53 ± 8.93 ^a^	700.67 ± 9.53 ^a^	599.13 ± 24.59	426.20 ± 18.69	441.07 ± 18.70 ^a^	3857.67 ± 11.09 ^a^
FI (gm)	Housing System	1–7 day	7–14 day	14–21 day	21–28 day	28–35 day	35–42 day	42–49 day	49–56 day	Total feed intake
	CH	311.00 ± 0.93	795.33 ± 3.07 ^a^	914.33 ± 11.93	1296.00 ± 13.93 ^b^	1699.00 ± 4.50 ^a^	1790.00 ± 8.11 ^a^	1788.00 ± 7.45 ^a^	1835.00 ± 3.78 ^a^	10,066.67 ± 218.35
	HY	312.20 ± 1.38	772.07 ± 4.01 ^b^	903.00 ± 10.41	1352.00 ± 9.82 ^a^	1618.00 ± 18.08 ^b^	1728.00 ± 10.61 ^b^	1792.00 ± 7.82 ^a^	1774.00 ± 5.17 ^b^	10,251.27 ± 32.30
	CHSP	308.13 ± 1.64	780.00 ± 5.75 ^b^	918.33 ± 4.75	1320.00 ± 11.83 ^ab^	1630.00 ± 1.89 ^b^	1792.67 ± 4.41 ^a^	1798.60 ± 7.03 ^a^	1827.00 ± 3.00 ^a^	10,374.73 ± 16.98
	HYSP	306.20 ± 2.19	755.60 ± 4.58 ^c^	922.00 ± 4.75	1335.67 ± 9.93 ^a^	1636.00 ± 7.04 ^b^	1673.00 ± 6.36 ^c^	1687.20 ± 16.61 ^b^	1781.00 ± 3.42 ^b^	10,096.67 ± 29.71
FCR (g feed/g gain)	Housing System	1–7 day	7–14 day	14–21 day	21–28 day	28–35 day	35–42 day	42–49 day	49–56 day	Final FCR
	CH	1.78 ± 0.01	2.18 ± 0.02 ^a^	2.14 ± 0.05	2.10 ± 0.03 ^a^	2.62 ± 0.03 ^a^	2.99 ± 0.05	4.32 ± 0.09	4.80 ± 0.11 ^a^	2.77 ± 0.06 ^a^
	HY	1.78 ± 0.02	2.06 ± 0.03 ^bc^	2.04 ± 0.05	2.04 ± 0.03 ^ab^	2.51 ± 0.08 ^a^	2.99 ± 0.06	4.22 ± 0.14	4.92 ± 0.20 ^a^	2.78 ± 0.02 ^a^
	CHSP	1.78 ± 0.02	2.14 ± 0.04 ^ab^	2.05 ± 0.04	2.07 ± 0.03 ^ab^	2.52 ± 0.03 ^a^	2.95 ± 0.08	4.34 ± 0.17	4.30 ± 0.15 ^b^	2.77 ± 0.01 ^a^
	HYSP	1.74 ± 0.02	1.97 ± 0.04 ^c^	2.03 ± 0.04	1.99 ± 0.03 ^b^	2.34 ± 0.04 ^b^	2.86 ± 0.12	4.07 ± 0.19	4.12 ± 0.15 ^b^	2.62 ± 0.01 ^b^

Means ± standard error; different superscripts within the same column signify significant difference (*p* ≤ 0.01). Body weight (BW); weight gain (WG); feed intake (FI); feed-conversion ratio (FCR). Closed house (CH); closed house with open yard (HY); closed house with swimming pool (CHSP); and closed house with swimming pool and yard (HYSP).

**Table 2:** Effect of different housing systems on carcass traits percentages of Pekin ducks.

Housing System	Dressing%	Liver%	Gizzard%	Heart%	Abdominal Fat%	Breast%	Thigh%	Shoulder%	Left Filet%
CH	60.00 ± 0.71 ^b^	4.14 ± 0.05 ^c^	4.00 ± 0.01	1.13 ± 0.02	2.36 ± 0.05 ^a^	28.30 ± 0.34 ^c^	11.72 ± 0.09 ^c^	7.20 ± 0.07	11.76 ± 0.22 ^b^
HY	61.00 ± 0.71 ^ab^	4.42 ± 0.06 ^ab^	4.02 ± 0.01	1.13 ± 0.04	2.11 ± 0.03 ^bc^	30.70 ± 0.49 ^b^	12.18 ± 0.07 ^b^	7.24 ± 0.07	12.02 ± 0.07 ^ab^
CHSP	61.20 ± 0.58 ^ab^	4.28 ± 0.04 ^bc^	4.00 ± 0.02	1.11 ± 0.05	2.17 ± 0.04 ^b^	29.60 ± 0.51 ^b^	12.21 ± 0.12 ^b^	7.19 ± 0.08	12.03 ± 0.06 ^ab^
HYSP	62.40 ± 0.51 ^a^	4.50 ± 0.04 ^a^	4.01 ± 0.01	1.11 ± 0.03	2.02 ± 0.03 ^c^	32.10 ± 0.33 ^a^	12.56 ± 0.12 ^a^	7.16 ± 0.07	12.22 ± 0.03 ^a^

Means ± standard; different superscripts within the same column signify significant difference (*p* ≤ 0.05 for dressing%) and (*p* ≤ 0.001). Closed house (CH); closed house with open yard (HY); closed house with swimming pool (CHSP); and closed house with swimming pool and yard (HYSP).

**Table 3:** Effect of different housing systems on blood biochemical parameters (mg/dL) and meat cholesterol and triacylglycerol (mg/dL of extract) of Pekin ducks.

Housing System	Albumin	Globulin	A/G Ratio	Total Protein	Total Lipids	Triacylglycerol	Cholesterol	Glucose	Meat Cholesterol	Meat Triacylglycerol
CH	1.88 ± 0.06 ^b^	1.67 ± 0.05 ^b^	1.13 ± 0.04 ^b^	3.55 ± 0.09 ^b^	607.18 ± 27.29 ^a^	146.18 ± 6.91 ^a^	161.64 ± 7.51 ^a^	226.00 ± 3.99 ^b^	143.80 ± 4.12 ^a^	131.80 ± 0.86 ^a^
HY	2.60 ± 0.02 ^a^	2.07 ± 0.04 ^a^	1.26 ± 0.02 ^ab^	4.67 ± 0.05 ^a^	484.20 ± 12.77 ^b^	115.01 ± 3.06 ^b^	132.82 ± 2.18 ^b^	243.00 ± 2.24 ^a^	132.80 ± 1.36 ^bc^	129.20 ± 0.58 ^a^
CHSP	1.91 ± 0.05 ^b^	1.69 ± 0.09 ^b^	1.14 ± 0.07 ^b^	3.59 ± 0.10 ^b^	582.52 ± 6.48 ^a^	142.88 ± 5.53 ^a^	161.14 ± 4.92 ^a^	227.40 ± 4.41 ^b^	139.60 ± 0.93 ^ab^	130.60 ± 0.93 ^a^
HYSP	2.62 ± 0.05 ^a^	1.95 ± 0.02 ^a^	1.35 ± 0.03 ^a^	4.56 ± 0.06 ^a^	491.74 ± 9.45 ^b^	118.91 ± 4.14 ^b^	134.91 ± 2.52 ^b^	243.40 ± 2.54 ^a^	130.00 ± 1.00 ^c^	126.60 ± 0.93 ^b^

Means ± standard error; different superscripts within the same column signify significant difference (*p* ≤ 0.001). Albumin/globulin ratio (A/G ratio). Closed house (CH); closed house with open yard (HY); closed house with swimming pool (CHSP); and closed house with swimming pool and yard (HYSP).

**Table 4:** Effect of different housing systems on cellular immunity of Pekin ducks.

Housing System	Phagocytic Index	Phagocytic Activity	Eosinophils%	Lymphocytes%	Heterophiles%	Basophiles%	Monocytes%
CH	1.53 ± 0.02 ^b^	16.30 ± 0.44 ^c^	7.42 ± 0.12 ^c^	34.48 ± 0.26	23.52 ± 0.21 ^d^	0.91 ± 0.09 ^b^	4.85 ± 0.08 ^b^
HY	1.64 ± 0.06 ^b^	17.34 ± 0.27 ^ab^	7.68 ± 0.08 ^bc^	34.66 ± 0.21	24.38 ± 0.25 ^c^	1.21 ± 0.06 ^a^	5.26 ± 0.05 ^a^
CHSP	1.61 ± 0.03 ^b^	16.74 ± 0.24 ^bc^	7.78 ± 0.15 ^b^	34.76 ± 0.40	25.00 ± 0.13 ^b^	1.08 ± 0.13 ^ab^	5.20 ± 0.17 ^a^
HYSP	1.77 ± 0.04 ^a^	17.84 ± 0.13 ^a^	8.12 ± 0.07 ^a^	35.28 ± 0.21	25.61 ± 0.15 ^a^	1.36 ± 0.07 ^a^	5.48 ± 0.09 ^a^

Means ± standard error; different superscripts within the same column signify significant difference (*p* ≤ 0.01). Closed house (C.H); closed house with open yard (HY); closed house with swimming pool (CHSP); and closed house with swimming pool and yard (HYSP).

**Table 5:** Effect of different housing systems on oxidative stress of Pekin ducks.

Housing System	MDA (nmol/mL)	GPx (U/gHb)	SOD (U/gHb)
CH	2.54 ± 0.05 ^a^	22.80 ± 0.73 ^a^	90.60 ± 0.93 ^a^
HY	1.90 ± 0.11 ^b^	18.80 ± 0.86 ^bc^	73.20 ± 1.93 ^b^
CHSP	2.10 ± 0.07 ^b^	20.80 ± 1.24 ^ab^	69.20 ± 8.73 ^b^
HYSP	1.58 ± 0.10 ^c^	16.80 ± 0.58 ^c^	52.60 ± 3.67 ^c^

Means ± standard error; different superscripts within the same column signify significant difference (*p* ≤ 0.001). Malondialdehyde (MDA); glutathione peroxidase (GPx); and superoxide dismutase (SOD). Closed house (CH); closed house with open yard (HY); closed house with swimming pool (CHSP); and closed house with swimming pool and yard (HYSP).

## Supplementary Materials

The following are available online at https://www.mdpi.com/2076-2615/10/3/410/s1, Figure S1: Closed house (CH); Figure S2: Closed house with open yard (HY); Figure S3: Closed house with swimming pool (CHSP); Figure S4: Closed house with swimming pool and yard (HYSP).
