# Supplementary material for: The Influences of Various Housing Systems on Growth, Carcass Traits, Meat Quality, Immunity and Oxidative Stress of Meat-Type Ducks
Source: Animals (Basel). 2020 Mar 2;10(3):410. doi: 10.3390/ani10030410 (PMC7143679; doi:10.3390/ani10030410)

# Supplementary material (Photos)

## 1. Closed house (CH)

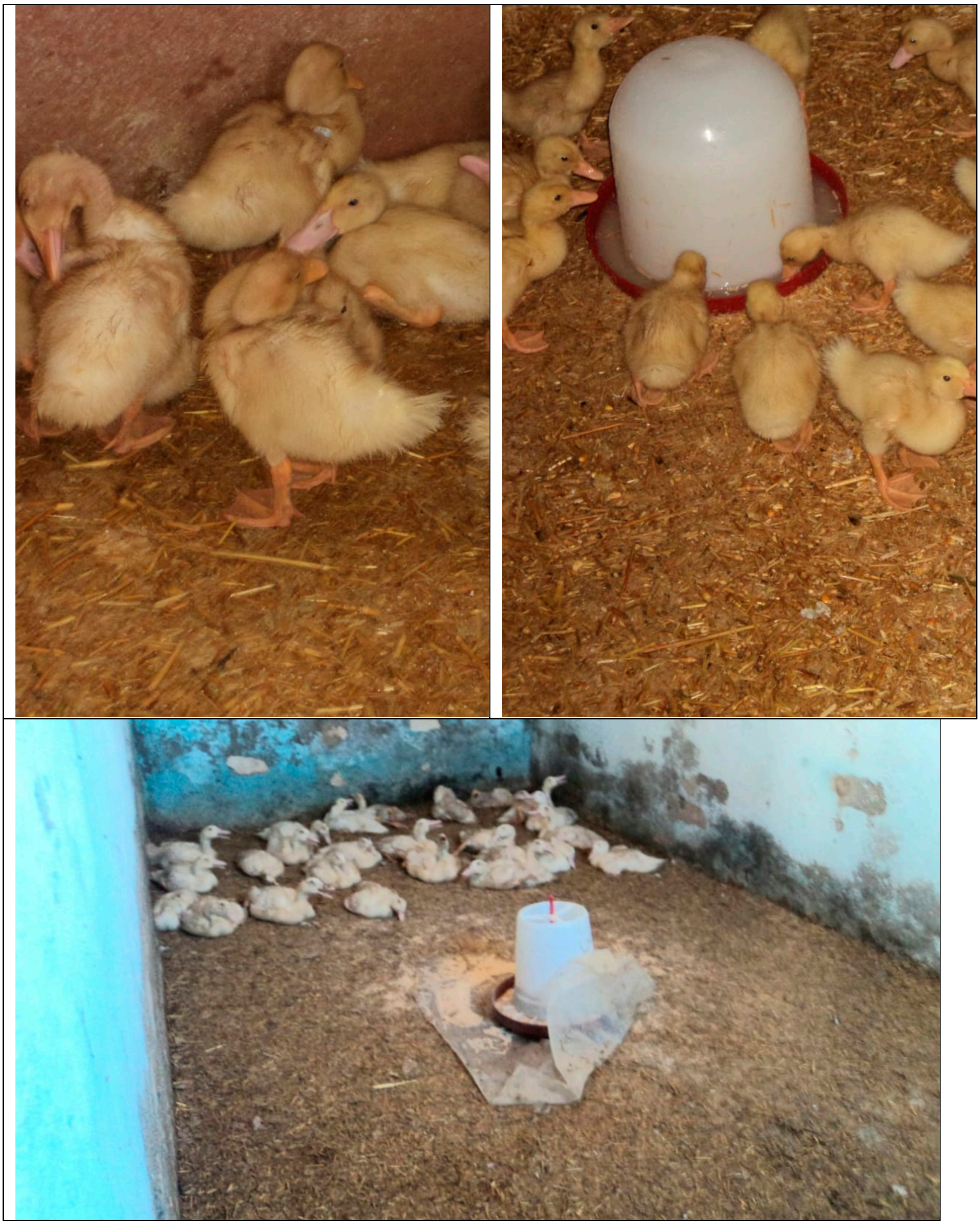

## 2. Closed house with open yard (HY)

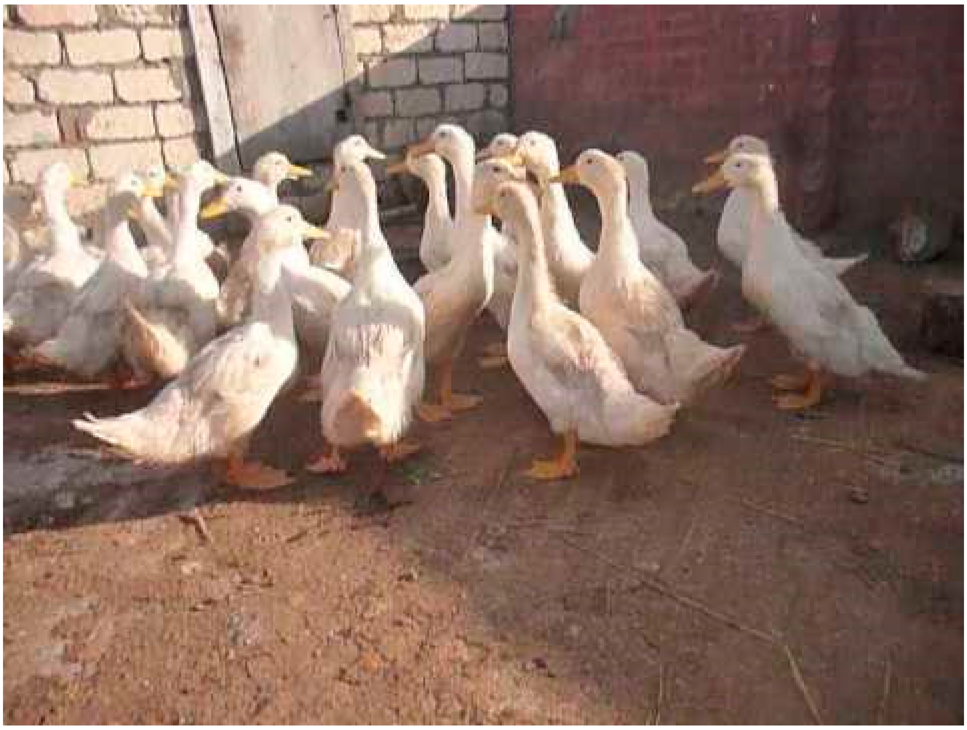

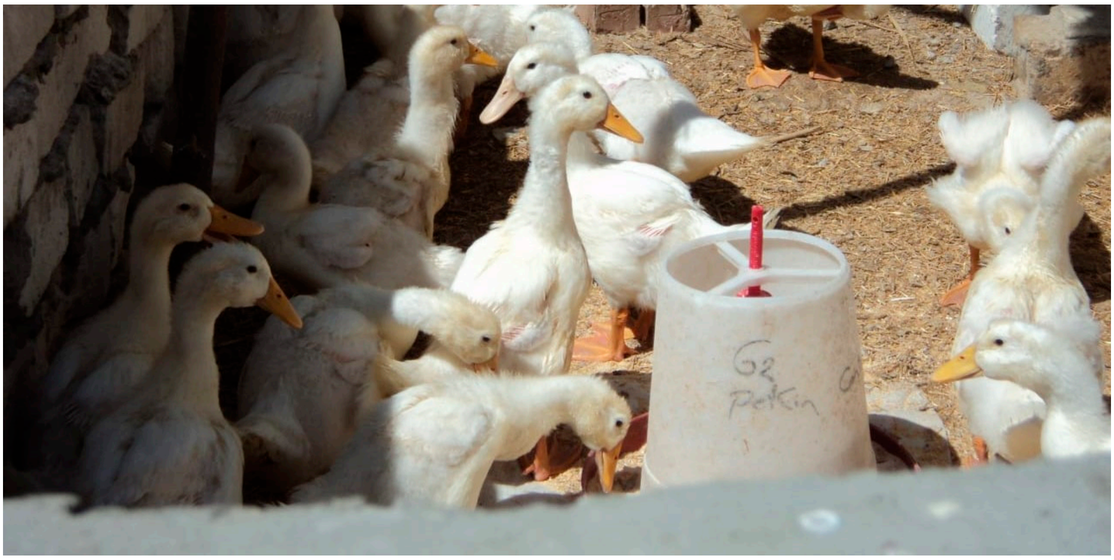

**3. Closed house with swimming pool (CHSP)**

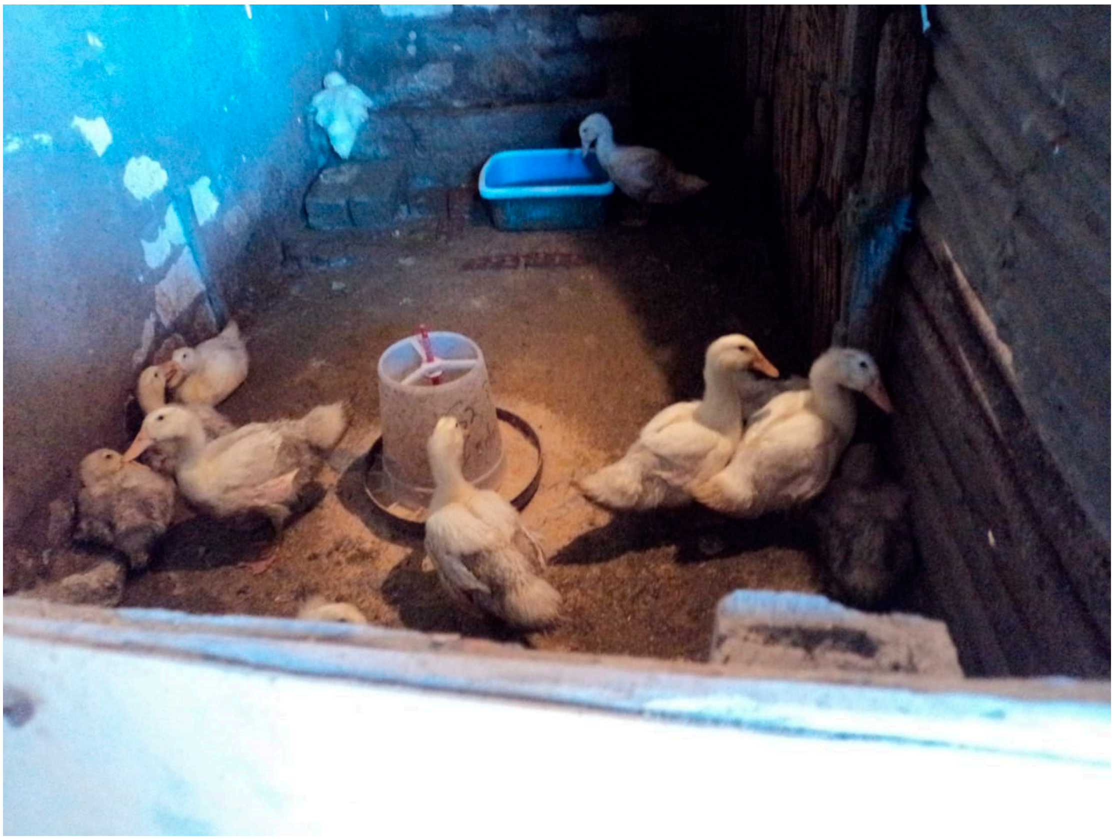

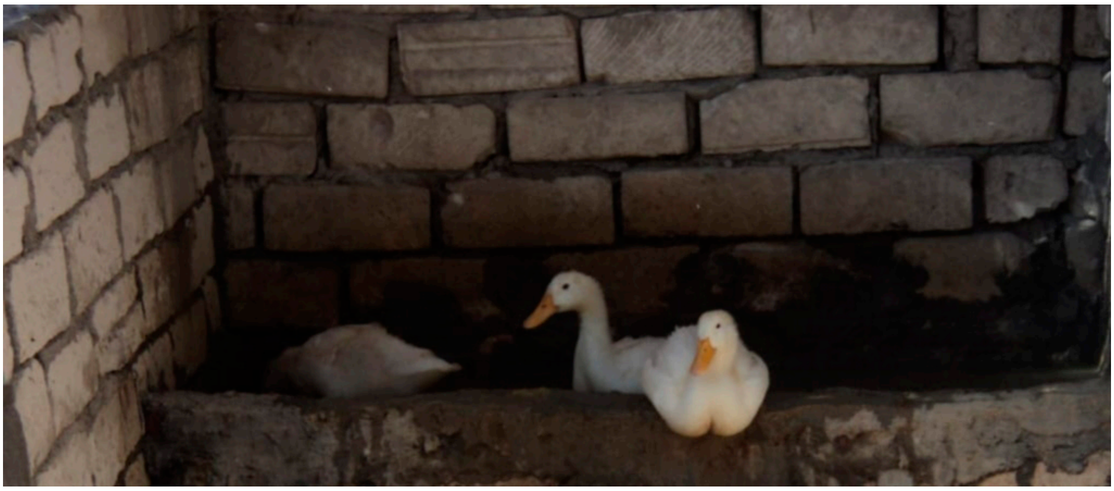

**4. Closed house with swimming pool and yard (HYSP)**

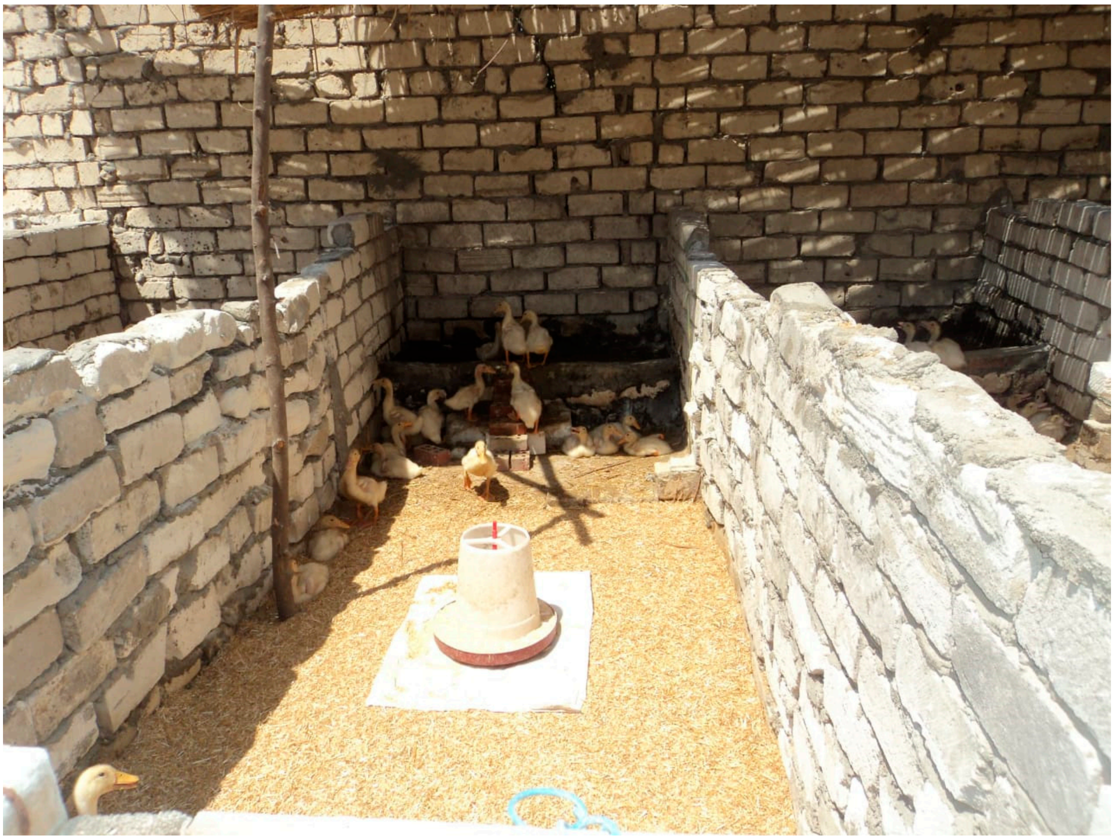

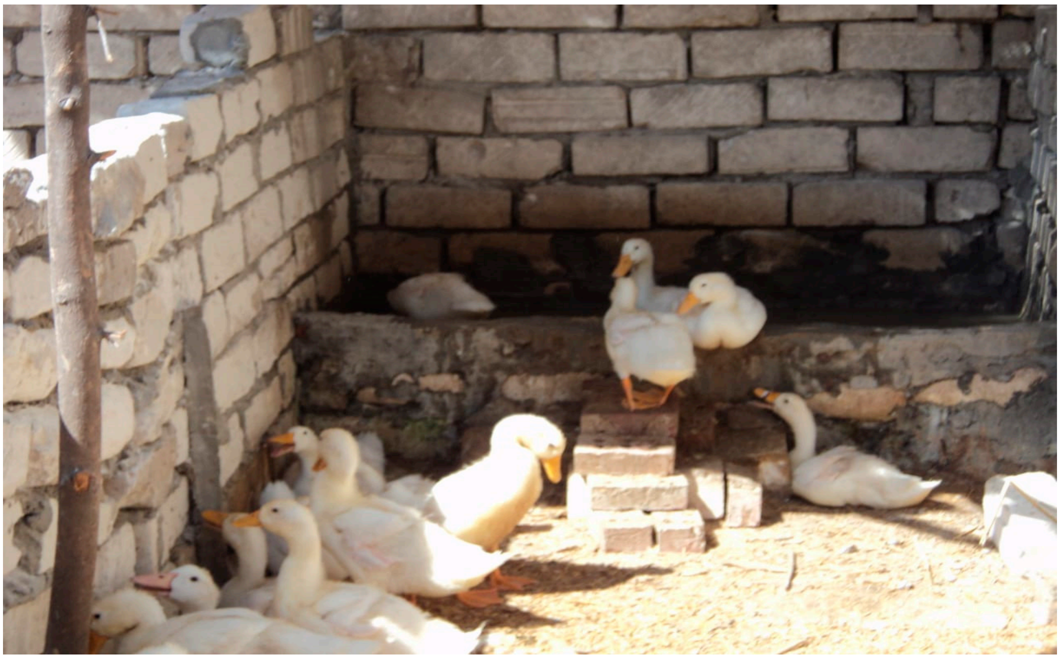

Supplement: Supplementary file 1 [file animals-10-00410-s001.pdf]
